# Variables associated with antibiotic treatment tolerance in patients with *Mycobacterium avium* complex pulmonary disease

**DOI:** 10.1186/s12931-024-02752-y

**Published:** 2024-03-11

**Authors:** Meghan Marmor, Husham Sharifi, Susan Jacobs, Kiana Fazeli, Stephen Ruoss

**Affiliations:** Division of Pulmonary, Allergy, and Critical Care Medicine, Stanford University School of Medicine, Stanford, CA United States of America

## Abstract

**Background:**

Treatment of *Mycobacterium avium* complex pulmonary disease (MAC-PD) involves prolonged courses of multiple antibiotics that are variably tolerated and commonly cause adverse drug reactions (ADR). The purpose of this retrospective, single-center study was to identify demographic and disease-related variables associated with significant ADRs among patients treated with antibiotics against MAC-PD.

**Methods:**

We reviewed all patients treated with antibiotic therapy for MAC-PD at a single center from 2000 to 2021. Patients were included if they met diagnostic criteria for MAC-PD, were prescribed targeted antibiotic therapy for any length of time and had their treatment course documented in their health record. We compared patients who completed antibiotics as originally prescribed (tolerant) with those whose antibiotic treatment course was modified or terminated secondary to an ADR (intolerant).

**Results:**

Over the study period, 235 patients were prescribed antibiotic treatment with their clinical course documented in our center’s electronic health record, and 246 treatment courses were analyzed. One hundred forty-three (57%) tolerated therapy versus 108 (43%) experienced ADRs. Among the 108 intolerant courses, 67 (63%) required treatment modification and 49 (46%) required premature treatment termination. Treatment intolerance was associated more frequently with smear positive sputum cultures (34% vs. 20%, *p* = 0.009), a higher Charlson Comorbidity Index (CCI) (4 vs. 6, *p* = 0.007), and existing liver disease (7% vs. 1%, *p* = 0.03). There was no between-group difference in BMI (21 vs. 22), fibrocavitary disease (24 vs. 19%), or macrolide sensitivity (94 vs. 80%). The use of daily therapy was not associated with intolerance (77 vs. 79%). Intolerant patients were more likely to be culture positive after 6 months of treatment (44 vs. 25%).

**Conclusions:**

Patients prescribed antibiotic therapy for MAC-PD are more likely to experience ADRs if they have smear positive sputum cultures at diagnosis, a higher CCI, or existing liver disease. Our study’s rate of early treatment cessation due to ADR’s was similar to that of other studies (20%) but is the first of its kind to evaluate patient and disease factors associated with ADR’s. A systematic approach to classifying and addressing ADRs for patients undergoing treatment for MAC-PD is an area for further investigation.

## Background

*Mycobacterium avium* complex (MAC) is the most common cause of nontuberculous mycobacterial (NTM) pulmonary disease in the United States and worldwide [1, 2]. The disease presents along a spectrum of severity from mild respiratory symptoms and minimal lung injury to debilitating symptoms and substantial structural and functional lung injury that reduces patients’ quality of life [3, 4]. Antibiotic treatment involves prolonged courses of multiple antibiotics that are variably tolerated, and adverse drug reactions (ADRs) including drug side effects, interactions and toxicities are commonplace [5–9].

ADRs including gastrointestinal symptoms, loss of visual acuity, loss of auditory acuity and leukopenia have been frequently described in association with guideline-based treatment and can complicate therapy [7, 10]. Prior studies have found that cytopenias and hepatotoxicity are some of the more common ADR’s and that while some may present early in a patient’s treatment course, others, like reactions to ethambutol, may present many months into treatment [11, 12]. Identifying which patients are more likely to suffer from ADRs is not well described. Small, observational studies have reported more frequent ADRs among elderly patients, but the strength of this association is not well understood [7, 13]. The purpose of this retrospective, single-center study was to determine the association of patient- and disease-level factors with treatment intolerance, defined as alteration or cessation of guideline-based antibiotic therapy for MAC pulmonary disease (MAC-PD).

## Methods

### Patients

We reviewed all patients seen in our hospital network’s pulmonary or infectious disease clinics from 2000 to 2021 treated for MAC-PD with guideline-based therapy. Patients with a confirmed diagnosis of cystic fibrosis by genetic and or sweat testing were excluded. A patient’s treatment course was included if they were 18 years of age or older at the time of therapy initiation, met the American Thoracic Society and Infectious Disease Society of America’s criteria for MAC-PD, were prescribed guideline-concordant antibiotic therapy for MAC-PD and had record of having taken the prescribed medication for any length of time. For patients who underwent multiple treatment courses for recurrent or relapsing disease, each treatment course was included independently. Patients were excluded if there was no record of their treatment course and outcomes. This study was approved by our institutional review board (IRB-60,666).

### Study design

The study was a retrospective, single-center cohort analysis. Clinical data were collected from medical records. Baseline clinical parameters were collected at the encounter when antibiotic therapy was first prescribed including sex, age, lung function as measured by forced expiratory volume in the first second, ethnicity, comorbid conditions, culture smear status, macrolide sensitivity, radiographic characteristics, and frequency of antibiotic administration. Radiographic abnormalities were classified according to chest computed tomography (CT) as either nodular bronchiectatic or fibrocavitary disease. Treatment courses were divided into two groups: those who tolerated therapy, completing guideline-concordant antibiotic course as prescribed [5, 14], and those who were intolerant, defined as having required either modification to their original treatment regimen or early cessation of therapy defined as termination before the total 12-month period of negative sputum cultures specifically due to an adverse drug reaction.

### Statistical analysis

Categorical baseline characteristics were reported by frequency and percent. Continuous characteristics are reported as a mean with standard deviations. Chi-squared tests were used to compare categorical variables, and Wilcoxon rank-sum tests were used to compare continuous variables. The Benjamini-Hochberg procedure was used for multiple comparisons. Univariate logistic regression was performed for individual patient characteristics and data with a single covariate in each case and with treatment tolerance as the dependent variable. Odds ratiods were calculated from the beta coefficient of each model. Multivariate logistic regression was performed with patient characteristics and data as covariates and with treatment tolerance of the dependent variable. Adjusted odds ratios were calculated from the beta coefficients of the composite model. Two-sided *p*-values < 0.05 were considered statistically significant.

## Results

### Baseline characteristics

During the study period, there were 329 treatment courses for MAC-PD identified. Among them, 246 met inclusion criteria with detailed reports of the treatment course available in the electronic health record. One hundred forty-two (57%) patients were tolerant of their prescribed therapy without adverse reactions versus 104 (43%) were intolerant.

As shown in Table 1, **75**% of participants were female, a mean age of 65 years, majority Caucasian (61%) with a baseline FEV_1_ of 79%. Daily therapy was prescribed initially for the majority of both groups (78%). Treatment intolerant courses had a higher rate of culture positivity at 6 months of therapy (43% vs. 25%, *p* < 0.001). Among the 104 intolerant treatment courses, 65 (63%) required treatment modification and 38 (37%) required premature termination. There was no significant difference in recurrent disease, defined as culture positivity within 12 months of completing treatment (20% vs. 24%), or mortality (9% vs. 12%) between groups. Similarly, there was no significant difference in treatment duration, where treatment for more than 18 months was similar between groups (33% vs. 29%).

Fifteen patients underwent more than one treatment course for MAC-PD. Among them, 10 tolerated all treatment courses; 3 patients tolerated their first treatment course but were later intolerant of subsequent treatment; and 2 patients were intolerant of their first treatment course but later tolerated the second treatment course.

**Table 1:** Baseline characteristics and treatment outcomes

	Tolerant	Intolerant	*P*-value
Treatment courses (n)	142	104	--
**Baseline Characteristics**			
Age (years (SD))	64.1 (12.5)	66.7 (12.0)	0.094
Sex (n, % female)	110 (77%)	79 (73%)	0.418
Baseline FEV1 (% predicted mean (SD))	80.5 (23.0)	76.4 (23.5)	0.22
Caucasian (n, %)	88 (62%)	61 (59%)	0.694
Asian (n, %)	36 (25%)	28 (27%)	0.896
Other ethnicity (non-Caucasian, non-Asian, n, %)	18 (13%)	15 (14%)	0.835
Body Mass Index (mean (SD))	21.5 (4.0)	22.3 (5.4)	0.121
Fibrocavitary Disease (n, %)	34 (24%)	20 (19%)	0.28
Smear positive (n, %)	28 (20%)	37 (34%)	**0.009**
Charlson Comorbidity Index (mean (SD))	4.8 (3.2)	6.1 (4.6)	**0.007**
Organ Transplant (n, %)	2 (1.4%)	7 (6.5%)	0.064
Liver Disease (n, %)	2 (1.4%)	8 (7%)	**0.032**
Chronic Kidney Disease Stage 3 or greater (n, %)	6 (4%)	6 (5.5%)	0.798
Immune Compromise, not HIV (n, %)	24 (17%)	21 (19%)	0.622
Diabetes (n, %)	7 (5%)	8 (8%)	0.532
Heart Failure (n, %)	10 (7%)	12 (11%)	0.320
Ocular Disease (n, %)	27 (19%)	22 (21%)	0.800
Connective Tissue Disease (n, %)	12 (9%)	15 (14%)	0.203
GERD (n, %)	36 (25%)	25 (24%)	0.931
**Disease Characteristics**			
Macrolide sensitive (n, %)	125 (88%)	102 (94%)	0.8721
Daily therapy (n, %)	109 (77%)	85 (79%)	0.816
Inhaled amikacin (n, %)	9 (6%)	8 (7%)	0.873
Amikacin liposomal inhalation suspension (n, %)	7 (5%)	15 (14%)	**0.033**
Parenteral amikacin on initiation (n, %)	8 (6%)	8 (7%)	0.932
**Outcomes**			
Mean treatment duration (months, range)	21 (9–54)	19 (0.5–55)	0.572
Culture positive at 6 months (n, %)	37 (25%)	45 (43%)	**< 0.001**
Recurrence within 12 months (n, %)	28 (20%)	17 (24%)	0.524
Death from any cause (n, %)	13 (9%)	13 (12%)	0.527

Data are presented as mean +- standard deviation or median, unless otherwise stated. Yrs: years; FEV1: forced expiratory volume in 1 s; Body mass index units measured in kg/m^2^; GERD: gastroesophageal reflux disease; CT pattern determined by the treating physician. Time to culture conversion calculated from treatment initiation until the first negative culture

### Adverse drug reactions and implicated antibiotics

Among intolerant patients, 19 (18%) underwent a dose reduction of the offending agent and among those on daily therapy, 9 (8.5%) were transitioned to thrice weekly treatment. Severe adverse drug reactions were relatively rare. Using Common Terminology Criteria for Adverse Events criteria, severe adverse drug events, defined as Grade 3 or greater, were seen in 13 intolerant and 3 tolerant treatment courses. Of the former, acute kidney injury from intravenous amikacin at treatment initiation was most common, while the latter was leukopenia attributed to azithromycin (Table 2**)**. Clarithromycin, a macrolide with a significant side effect profile, accounted for intolerance in 2 cases. Rifabutin, a rifamycin with a significant side effect profile, accounted for intolerance in 8 cases.

As reported in Table 2, gastrointestinal disturbances were the most commonly reported side effect in both groups. Intolerant patients suffered disproportionately with a greater burden of GI upset, fatigue, visual disturbances, and rashes. Rifamycins dominated as the most commonly implicated antibiotic for adverse drug reactions most commonly due to drug-drug interactions.

**Table 2:** Adverse drug reactions and implicated antibiotics

Adverse Drug Reactions	Tolerant	Intolerant
Common Terminology Criteria for Adverse Events Grade 3 or greater^1^ (Total)	3 (2%)	13 (13%)
Gastrointestinal symptoms	34 (23%)	58 (55%)
Rash	4 (3%)	9 (8.5%)
Fatigue	5 (3%)	23 (22%)
Cough, dysphonia, or bronchospasm	4 (3%)	4 (4.7%)
Visual disturbance	2 (1%)	11 (10%)
Auditory disturbance	1 (0.7%)	5 (5%)
Acute kidney injury	0	4 (4%)
Hemoptysis	0	1 (1%)
Anaphylaxis	0	1 (1%)
Other	6 (4%)	8 (8%)
**Implicated Antibiotic**		
Macrolides	12 (8%)	21 (19%)
Rifamycins	4 (3%)	46 (43%)
Ethambutol	3 (2%)	22 (20%)
Amikacin liposomal inhalation suspension	4 (3%)	11 (10%)
Parenteral Amikacin	0	5 (5%)

1: Common Terminology Criteria for Adverse Events [CTCAE] Grade 3 is defined as severe or medically significant but not immediately life-threatening; hospitalization or prolongation of hospitalization indicated; disabling; limiting self-care

### Treatment intolerance

**Fig. 1:**
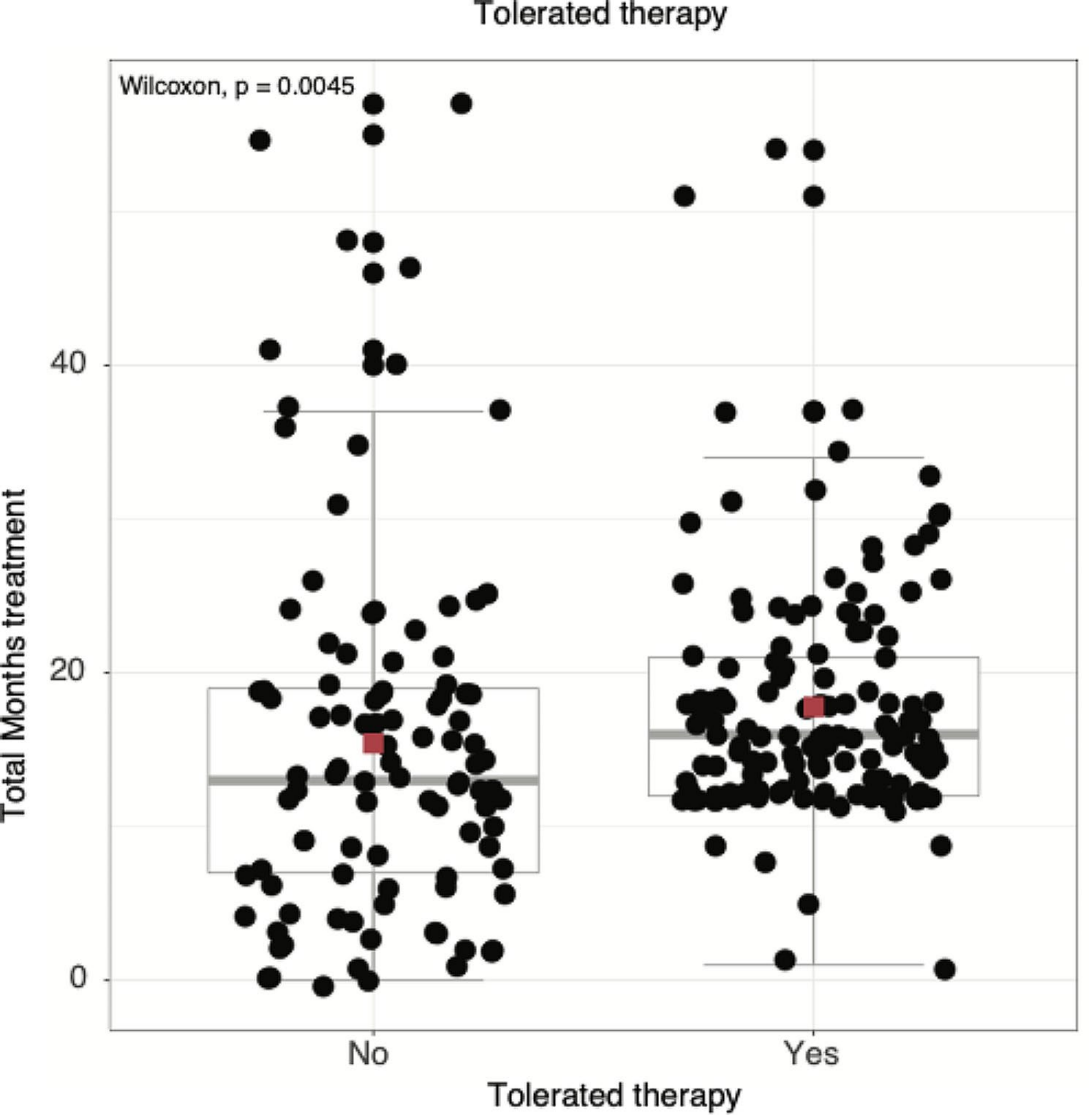
Associations between treatment tolerance and treatment time. Red square is mean, horizontal grey line is median, box is interquartile range. Each dot represents a treatment course for a patient

In a univariate analysis, intolerance was associated with older age, smear positive cultures, a higher Charlson Comorbidity Index (CCI), pre-existing liver disease, receipt of amikacin liposomal inhalation suspension, and the receipt of a solid organ or bone marrow transplant (Table 3). Multivariate regression with risk adjustment attenuated the signal for all variables -- other than smear status positivity -- though a trend to significance was maintained. Figure 1 shows that persons who were intolerant of treatment had a wider distribution of treatment times.

**Table 3:** Univariate and multivariate logistic regression for intolerance of therapy

	Univariate Logistic Regression		Multivariate Logistic Regression	
	OR (95% CI)	*P*-Value	Adjusted OR (95% CI)	*P*-Value
Age (yrs)	1.02 (1.00–1.05)	**0.05**	1.02 (0.99–1.05)	0.089
BMI	1.04 (0.98–1.10)	0.20	1.06 (0.99–1.13)	0.078
CCI	1.10 (1.03–1.18)	**0.007**	1.07 (0.99–1.17)	0.095
Liver disease	5.87 (1.43–39.5)	**0.027**	4.66 (1.00–33.27)	0.078
Smear status	2.41 (1.33–4.40)	**0.004**	2.52 (1.32–4.87)	**0.005**
Solid organ transplant	5.01 (1.20–34.6)	**0.046**	4.54 (0.90–34.10)	0.088
Use of Arikayce	3.94 (1.43–12.7)	**0.012**	2.96 (0.91–10.52)	0.076

## Discussion

In this study, we investigated patient- and disease-level factors associated with tolerance of antibiotic therapy for MAC-PD. Our study included 246 treatment courses, where more than half tolerated treatment and less than half did not. Our rate of intolerance was similar to previously reported rates with 104 (42%) of treatment courses impacted by a significant ADR, where 38 (15%) required premature termination of therapy [6, 15, 16]. We found an association between antibiotic intolerance and smear positivity on sputum culture, and a trend towards significance in association with advanced age, high burden of comorbid illness, the use of amikacin liposomal inhalation suspension, pre-existing liver disease and receipt of a solid organ or bone marrow transplant. Antibiotic treatment intolerance was most commonly due to patient-reported side effects. As expected, these individuals had worse clinical outcomes including a greater rate of persistent culture positivity.

Sustained culture conversion rates have been reported in the literature as high as 86% in those treated with guideline-based therapy [8, 17]. Unfortunately, ADRs from multidrug antibiotic regimens are commonplace, as frequent as 40% [18]. ADRs pose a serious challenge for MAC-PD treatment because they often necessitate therapy modifications and potentially less efficacious regimens or cessation of therapy all together.

Respiratory culture smear positivity at the time of treatment initiation was associated with intolerance. The authors hypothesize that this association may exist because smear positivity suggests a greater pathogen burden and potentially a more significant inflammatory reaction with antibiotic exposure. Previous studies of MAC-PD note that smear-positive respiratory cultures are associated with disease progression, and guidelines recommend initiating antibiotic therapy in individuals with this disease feature once the diagnosis is established [19, 20]. Interestingly, fibrocavitary disease, often suggested as a clinical feature associated with greater pathogen burden, was not associated with treatment intolerance. Both smear positivity and the presence of a cavity carry the potential for longer treatment courses and a cavity necessitates a more intensive initial therapy regimen. The authors question if the relatively impenetrable granulomatous inflammation of fibrocavitary disease serves to contain the pathogen, such that when an antibiotic is present, the host is ’protected’ from a more exaggerated inflammatory response during pathogen killing.

Age appeared to approach a statistically significant association with treatment intolerance. Smaller observational studies have noted a greater burden of ADRs in geriatric patients and some experts would argue for cautious dose administration in this population [13, 21]. Clinicians are sometimes reluctant to treat the elderly for MAC-PD out of concern for ADRs, citing relevant issues including the frequently greater burden of comorbid disease in older individuals, impaired drug metabolism owing to reduced renal or hepatic function, and risk of drug interactions owing to polypharmacy. However, chronological age does not directly translate into physiologic age, where two elderly patients of the same age may have very different life expectancies and functional statuses. Patients in our study received treatment because presumably the prescribing clinician felt that they were appropriate for treatment. While age approached a statistically significant association with intolerance, the authors argue that age alone should not dictate a patient’s candidacy for antibiotic therapy.

Burden of comorbid illness, as measured by the Charlson Comorbidity Index (CCI), approached a statistically significant association with intolerance. The CCI prediction model estimates a patient’s 10-year survival rate, where a higher score predicts a lower survival rate. On the whole, our center’s patient cohort had low CCI scores, similar to other studies [22–24]. These lower CCI scores likely reflect the fact that prescribing clinicians expected patients to live many years beyond their diagnosis, and 12 or more months of antibiotics was clinically appropriate. We hypothesize that a person with a higher CCI is more likely to experience ADRs because they are more likely to be taking other medications creating a risk of drug interactions and may have impaired drug metabolism owing to impaired organ function. Larger prospective clinical trials are needed to further evaluate this association.

Pre-existing liver disease, specifically cirrhosis and receipt of a solid organ transplant appeared to approach a statistically significant association with treatment intolerance. Cirrhosis can reduce a drug’s protein binding and porto-systemic shunting can impair first-pass metabolism of an orally administered medication. Taken together, this has the potential to substantially increase drug levels of an already hepatotoxic medication like rifampin or azithromycin and may predispose to intolerance. Similarly, organ transplant recipients are subject to variable antibiotic pharmacokinetics due to interactions with MAC-PD antibiotics and the cytochrome p450 metabolism of immune modulatory and prophylactic medications; these interactions may lead to increased risk of organ rejection or increase the risk of drug toxicities. Larger prospective studies are needed to better elucidate the strength of these associations.

Receipt of ALIS appeared to approach a statistically significant association with treatment intolerance. For our cohort, ALIS was exclusively used as an add-on therapy for treatment refractory cases where patients did not successfully culture convert after 6 months of guideline-based therapy [25]. The use of ALIS most commonly resulted in bronchospasm and cough, but also lead to hemoptysis in one patient and epistaxis in another. The authors posit that the use of ALIS hallmarks patients with treatment refractory disease who may have longer treatment courses with greater antibiotic exposure and are, therefore, more likely to experience adverse drug reactions.

Rifamycins were the most commonly implicated drug class in the intolerant group. This finding adds impetus to the ongoing and separate clinical study comparing MAC-PD treatment with and without rifamycins [26]. If therapy outcome excluding rifamycins is non-inferior to standard treatment, the removal of rifamycins from standard therapy for MAC-PD could substantially reduce ADRs for this population and potentially improve net therapy compliance and treatment outcomes.

Interestingly, lower BMI, while associated with greater mortality risk [27–29], was not associated with treatment intolerance. While weight-based antibiotic dose adjustments are incorporated in MAC-PD therapy, underweight patients may potentially receive higher exposures to antibiotics like macrolides during their therapy. However, our findings did not find an association between low BMI and intolerance.

Importantly, our data reveal that the use of daily antibiotic therapy was not associated with treatment intolerance. Two prior retrospective case series reported significantly greater drug intolerance (and need for change in drug regimen) in patients receiving daily therapy [8, 30], and those studies formed the central basis for the consensus guideline recommendation to treat uncomplicated PAC-PD with thrice weekly therapy. Our data are not concurrent with those prior case series. It is not known what clinical factors might explain the differences between our report and prior case series reports regarding tolerance of daily therapy, but it could relate to differences in close monitoring and treatment management, which are important factors in achieving best outcomes in these complex disease treatment circumstances. And perhaps also related to our careful therapy monitoring and management, treatment duration was not associated with intolerance in our study; overall, the treatment tolerant group had a greater and longer drug exposure compared to those who were intolerant. Whatever the explanations, our data argue for a possible reappraisal of the guideline recommendations for thrice weekly therapy for uncomplicated primary MAC-PD.

MAC-PD is known to recur or relapse, requiring multiple treatment courses over a patient’s lifetime. Of the 15 patients who underwent multiple treatment course in our study, the majority tolerated each treatment course without difficulty and 2 who struggled with intolerance during their first treatment course later tolerated their second. Presumably, clinicians could anticipate ADR’s from the first treatment course and modify their prescribing practice for the second. Tolerance of repeat therapy for recurrence or relapse is an area for further investigation, but our data suggest that repeating therapy later in life when a person is subject to older age, frailty, comorbid disease and polypharmacy does not guarantee that they will be intolerant.

Almost half of our cohort was treatment intolerant, similar to reported rates from other studies [31]. Most were able to continue with modifications, but a substantial proportion had to stop all together. The NTM-LD treatment guidelines acknowledge the high frequency of ADRs and offer clinicians recommendations to improve antibiotic tolerance including the favored use of azithromycin over clarithromycin for macrolide-susceptible strains as well as thrice weekly rather than daily therapy for nodular bronchiectatic disease. More recent publications have offered clinicians suggested substitutions for poorly tolerated guideline-based antibiotics [32]. Given the high rate and frequency of treatment intolerance that may compromise outcomes, developing a structured framework for clinicians to better triage and successfully modify therapy in response to ADRs is an area for future growth within the field.

Our study had several limitations. First, data collection was retrospective where clinical data and reported tolerance were performed based on clinician practice and patient report rather than systematically, including reporting of adverse drug reactions, surveillance sputum collection and other clinical outcomes. Second, our small, single-center cohort limits our ability to detect significant differences between groups and generalizability of our findings. For these reasons, a multi-center, prospective study is required to further clarify factors that predict treatment intolerance.

## Conclusion

The present study demonstrated that adverse drug reactions are common for patients undergoing NTM-LD antibiotic therapy. Smear positive sputum cultures at the time of diagnosis was associated with treatment intolerance. Lower BMI and daily therapy were not associated with intolerance. Given the high rate of treatment intolerance that may compromise outcomes, developing a structured framework for clinicians to better triage and successfully modify therapy in response to ADRs is an area for future growth within the field.

## Data Availability

The data for this study was from our institution’s electronic health record and is not available outside of our institution.
